# COVID-19 pandemic in BRICS countries and its association with socio-economic and demographic characteristics, health vulnerability, resources, and policy response

**DOI:** 10.1186/s40249-021-00881-w

**Published:** 2021-07-08

**Authors:** Jingmin Zhu, Wenxin Yan, Lin zhu, Jue Liu

**Affiliations:** 1 Department of Economics, University of Birmingham, Birmingham, B15 2TT UK; 2 Department of Epidemiology and Biostatistics, School of Public Health, Peking University, Haidian District, No. 38, Xueyuan Road, Beijing, 100191 China; 3 Center for Primary Care and Outcomes Research, School of Medicine, Center for Health Policy, Freeman Spogli Institute for International Studies, Stanford University, 450 Jane Stanford Way, Stanford, CA 94305-2004 USA; 4 Institute for Global Health and Development, Peking University, No. 5 Yiheyuan Road, Haidian, Beijing, 100871 China; 5 National Health Commission Key Laboratory of Reproductive Health, Peking University, No. 38, Xueyuan Road, Haidian, Beijing, 100191 China

**Keywords:** COVID-19, BRICS countries, Policy response, Associated factors

## Abstract

**Background:**

Little attention has been paid to the comparison of COVID-19 pandemic responses and related factors in BRICS (Brazil, Russia, India, China, and South Africa) countries. We aimed at evaluating the association of daily new COVID-19 cases with socio-economic and demographic factors, health vulnerability, resources, and policy response in BRICS countries.

**Methods:**

We conducted a cross-sectional study using data on the COVID-19 pandemic and other indicators of BRICS countries from February 26, 2020 to April 30, 2021. We compared COVID-19 epidemic in BRICS countries and analyzed related factors by log-linear Generalized Additive Model (GAM) models.

**Results:**

In BRICS countries, India had the highest totally of confirmed cases with 18.76 million, followed by Brazil (14.45 million), Russia (4.81 million), and South Africa (1.58 million), while China (0.10 million) had the lowest figure. South Africa had the lowest rate of administered vaccine doses (0.18 million) among BRICS countries as of April 30, 2021. In the GAM model, a 1 unit increase in population density and policy stringency index was associated with a 5.17% and 1.95% growth in daily new COVID-19 cases (*P* < 0.001), respectively. Exposure–response curves for the effects of policy stringency index on daily new cases showed that there was a rapid surge in number of daily new COVID-19 cases when the index ranged from 0 to 45. The number of infections climbed slowly when the index ranged from 46 to 80, and decreased when the index was above 80 (*P* < 0.001). In addition, daily new COVID-19 cases (all *P* < 0.001) were also correlated with life expectancy at birth (-1.61%), extreme poverty (8.95%), human development index (-0.05%), GDP per capita (-0.18%), diabetes prevalence (0.66%), proportion of population aged 60 and above (2.23%), hospital beds per thousand people (-0.08%), proportion of people with access to improved drinking water (-7.40%), prevalence of open defecation (0.69%), and annual tourist/visitor arrivals (0.003%), after controlling other confounders. Different lag structures showed similar results in the sensitivity analysis.

**Conclusions:**

Strong policy response is crucial to control the pandemic, such as effective containment and case management. Our findings also highlighted the importance of reducing socio-economic inequalities and strengthening the resilience of health systems to better respond to public health emergencies globally.

**Graphic abstract:**

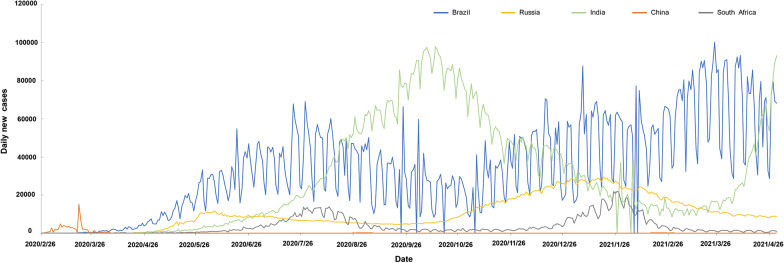

## Background

Coronavirus disease 2019 (COVID-19) has become a global public health issue. Figures from the World Health Organization (WHO) showed that more than 150.99 million COVID-19 cases have been confirmed worldwide, with over 3.17 million deaths as of May 1, 2021 [1]. Comprehensive measures have been taken across the world to curb the spread of the new severe acute respiratory syndrome coronavirus (SARS-CoV-2). The COVID-19 pandemic has affected countries in different ways [2]. BRICS constitute a political and economic grouping of countries (Brazil, Russia, India, China, and South Africa) undergoing rapid economic development, making up almost half of the world’s population. As of April 30, the number of SARS-CoV-2 infections in BRICS countries has reached 39.77 million, accounting for more than a quarter (26.3%) of the global total.

Previous analyses have focused on experiences and challenges in a specific country or globally [3–9]. Rocha et al. [6] found that existing socioeconomic inequalities rather than other risk factors have affected the course of COVID-19 epidemic in Brazil. Jefferies et al. [10] reported that New Zealand's strong response to COVID-19 resulted in low relative burden of disease, low levels of population disease disparities from Feb 2 to May 13, 2020. Romaniuk et al. compared health system outcomes in the BRICS countries during 2000–2017 and found a weak correlation with economic background [11]. Studies are needed to determine how exactly socio-economic characteristics, resources, and policy affect health system outcomes [11].

The influence of BRICS countries in the international arena has risen enormously in recent decades [12]. However, little attention has been paid to the comparison of COVID-19 pandemic responses and related factors in BRICS countries, despite their increasing global significance as individual countries and as an economic grouping [13]. Comparing COVID-19 situations and exploring its related factors in BRICS countries are important to better understand the spread of SARS-CoV-2 and pandemic control. This work is aimed at evaluating the association of daily new COVID-19 cases with socio-economic and demographic factors, health vulnerability, resources, and policy response among BRICS countries through a comparative study.

## Methods

### Data collection

This study analyzed data on the COVID-19 pandemic and other indicators of BRICS countries (Brazil, Russia, India, China, and South Africa) as of April 30, 2021. The indicators were selected based on availability and authenticity of data, as well as the assumption that they are the best available direct indicators related to the outcomes [6, 14, 15]. All data used in this study were collected from the public databases of Our World in Data, WHO, United Nations, World Bank, and other references.

### Data on response and COVID-19 outcomes

We used daily COVID-19 data from Our World in Data (https://ourworldindata.org/coronavirus-source-data) [16], which collected different sets of data related to COVID-19 from multiple sources globally. The number of COVID-19 cases in the Our World in Data database was sourced from Johns Hopkins University dashboard, which has been publishing updates on confirmed cases and deaths for all countries since January 22, 2020 [17]. The total administered vaccine doses, total confirmed COVID-19 cases, and transmission classification as of April 30, 2021 were derived from WHO (https://covid19.who.int/). In terms of transmission classification, WHO has defined four transmission scenarios for COVID-19, including no cases (countries with no cases), sporadic cases (countries with one or more cases, imported or locally detected), clusters of cases (countries experiencing cases, clustered in time, geographic location, and/or by common exposure), and community transmission (countries experiencing larger outbreaks of local transmission) [1].

In previous studies, a daily policy stringency index was used to reflect the level of policy response [6, 18–20]. The national stringency index, compiled from a global database of pandemic policies (Oxford COVID-19 Government Response Tracker, OxCGRT), addresses the need for continuously updated, readily usable and comparable information on policy measures [18]. This database enables policymakers and researchers to explore the empirical effects of policy responses on the spread of COVID-19. The policy stringency index ranged from a score of 0 to 100. The higher the score, the higher the level of policy response.

### Data on socio-economic and demographic characteristics, health vulnerability and resources

The data on socio-economic and demographic characteristics (including total population, population density, life expectancy at birth, extreme poverty, human development index and GDP per capita), health vulnerability (including diabetes prevalence, cardiovascular death rate and proportion of population aged 60 and above) as well as health system resources (hospital beds per thousand people) of BRICS countries were sourced from the public database of the United Nations (UN) (http://data.un.org/) and World Bank (https://data.worldbank.org/). The UN’s public tourism and transport database was also used to compile tourist/visitor arrivals to reflect population mobility.

Given that water and sanitation resources might also affect the spread of COVID-19, we included the proportion of people with access to improved drinking water and prevalence of open defecation as potential indicators [6, 21]. The proportion of people with access to improved drinking water was derived from previous studies in BRICS countries [21]. As one of the indicators of the sustainable development goals, the prevalence of open defecation was obtained from the latest UN report (https://www.sdg6data.org/indicator/6.2.1a).

### Statistical analysis

We used descriptive statistics and estimated Pearson coefficients for bilateral correlations between socio-economic and demographic factors, health vulnerability, resources, policy response to the COVID-19 pandemic and daily new cases in BRICS countries.

A log-linear Generalized Additive Model (GAM) model was used to analyze the associations between the expectation of the response variable (daily new cases) and the nonparametric predictor variables (such as policy stringency index) in this study. The GAM model is a combination of the generalized linear model and the additive model, which has been widely used in the research of infectious diseases using a connection function to establish the relationship between response variable and predictor variables [22–24]. The formula of the model is defined as follows:

$$logY_{t} = \alpha + \beta _{1} Var_{{1t}} + \beta _{2} Var_{{2t}} + \beta _{2} Var_{{3t}} + \ldots + {\text{ }}\beta _{n} Var_{{nt}} + + s(PSI,{\text{ }}df{\text{ }}) + Country_{i} + Week + day_{t}$$

In this formula, *log*(*Y*_*t*_) is the log-transformed of the number of daily new COVID-19 cases on day_t_; *α* is the intercept; *df* is the degree of freedom; *Var*_*1t*_ ~ *Var*_*nt*_ represent the variables of socio-economic and demographic factors (population density, life expectancy at birth, extreme poverty, human development index, GDP per capita), health vulnerability (diabetes prevalence, and proportion of population aged 60 and above), health system, water and sanitation resources (hospital beds per thousand people, proportion of people with access to improved drinking water, prevalence of open defecation), and population mobility (tourist/visitor arrivals). *PSI* is the policy response (policy stringency index) to the COVID-19 pandemic on day_t_; *s()* refers to a thin plate spline function based on the penalized smoothing spline to fit the long-term trend of the time. *Country*_*i*_ is a categorical variable for BRICS countries. Week is a categorical variable indicating the date of the week to control the day of the week effect, because the number of daily new cases might always be higher on one day of the week (such as Monday) as reported in previous studies [23], while *day*_*t*_ is the number of days with COVID-19 cases in a country to capture the day fixed effect. The econometric problems (normality, multicollinearity, autocorrelation) had been checked and we didn't find any problems. Since the effect of related factors might last several days and there is an incubation period of COVID-19 from exposure to infection, we also examined the associations with different lag structures (lag7 and lag14) in the sensitivity analysis. Lag7 refers to a lag of 7 days (median incubation period) and lag14 refers to a lag of 14 days (max incubation period) of COVID-19. GAMs were implemented via the “mgcv” package (version 1.8–28) of R software (version 3.5.2, Vienna, Austria). *P*-value < 0.05 was considered as statistically significant.

## Results

### Comparison of COVID-19 epidemic in BRICS countries

**Fig. 1:**
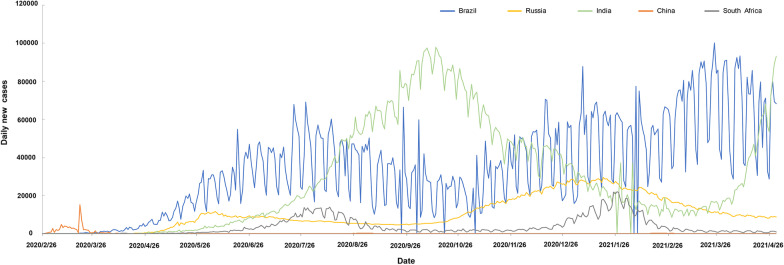
Trends of daily new cases in BRICS countries. *BRICS* Brazil, Russia, India, China, and South Africa

In BRICS countries, India had the highest totally of confirmed cases with 18.76 million, followed by Brazil (14.45 million), Russia (4.81 million), and South Africa (1.58 million), while China (0.10 million) had the lowest figure as of April 30, 2021 (Table 1). Daily new cases were also highest in India (386 452), followed by Brazil (79 726), Russia (8731), South Africa (1086), and China (33). The total number of administered vaccine doses was highest in China (147.73 million), followed by India (147.73 million), Brazil (35.53 million), and Russia (18.15 million). South Africa had the lowest rate of administered vaccine doses (0.18 million) among BRICS countries as of April 30, 2021. Both Brazil and South Africa were in community transmission mode, while there were clusters of cases in the three other countries. There were differing trends in daily new cases among BRICS countries, but all countries had undergone at least two COVID-19 waves from February 2020 to April 2021 (Fig. 1).

**Table 1:** Comparison of socio-economic and demographic characteristics, health vulnerability and resources, and policy response to COVID-19 pandemic in BRICS countries

Characteristics	BRICS countries				
	Brazil	Russia	India	China	South Africa
*Socio-economic and demographic characteristics*					
Total population (millions)	212.56	145.93	1380.00	1439.32	59.31
Population density (people per km^2^)	25.04	8.82	450.42	147.67	46.75
Life expectancy at birth (years)	75.88	72.58	69.66	76.91	64.13
Extreme poverty	3.40	0.10	21.20	0.70	18.90
Human development index	0.77	0.82	0.65	0.76	0.71
GDP per capita (current USD)	14 103.45	24 765.95	6426.67	15 308.71	12 294.88
*Health vulnerability*					
Diabetes prevalence (%)	8.11	6.18	10.39	9.74	5.52
Cardiovascular death rate (%)	177.96	431.30	282.28	261.90	200.38
Population aged 60 and above (%)	14.05	22.41	10.12	17.35	8.54
*Health system resources*					
Hospital beds per thousand people	2.20	8.05	0.53	4.34	2.32
*Water and sanitation*					
Access to improved drinking water (%)	96.90	97.00	90.70	91.50	91.40
Prevalence of open defecation (%)	1.13	0.00	25.73	0.00	1.45
*Population mobility*					
Tourist/visitor arrivals (thousands per year)	6621	24 551	17 423	62 900	10 472
*Policy response to COVID-19 pandemic as of April 30, 2021*					
Policy stringency index	70.83	42.13	69.91	78.24	48.15
Total administered vaccine doses (millions)	35.53	18.15	147.73	211.22	0.18
Daily new cases	79 726	8731	386 452	33	1086
Total confirmed cases (millions)	14.45	4.81	18.76	0.10	1.58
Transmission classification	Community transmission	Clusters of cases	Clusters of cases	Clusters of cases	Community transmission

*BRICS* Brazil, Russia, India, China, and South Africa, *GDP* Gross domestic product, *COVID-19* Coronavirus disease 2019, *USD* United States Dollars

### Comparison of socio-economic, demographic characteristics and other indicators in BRICS countries

Table 1 shows the comparison of socio-economic, demographic characteristics and other indicators in BRICS countries. Brazil had the lowest tourist/visitor arrivals. Russia topped the human development index and had the least stringent policy. In contrast, China had the largest tourist/visitor arrivals and strictest policy response. On the other hand, India had the highest prevalence rate in open defecation, while its GDP per capita, hospital beds per thousand people and proportion of people with access to improved drinking water were the lowest. South Africa had the smallest population and the lowest proportion of population aged 60 and above.

### Correlation coefficients between daily new cases and related indicators

Daily new COVID-19 cases were correlated with socio-economic and demographic disadvantages, health and resource vulnerabilities, population mobility, and policy response (Table 2). There was also a positive correlation among daily new COVID-19 cases with population density, extreme poverty, proportion of population aged 60 and above, prevalence of open defecation and policy stringency (*P* < 0.001). On the other hand, life expectancy at birth, human development index, GDP per capita, hospital beds per thousand people, and access to improved drinking water were negatively correlated with daily new COVID-19 cases (*P* < 0.001).

**Table 2:** Correlation coefficients among socio-economic and demographic characteristics, health vulnerability and resources, policy response and daily new COVID-19 cases in BRICS countries

Variables	Daily new cases	
	Coefficients	*P*-value
Population density (people per km^2^)	0.04	0.072
Life expectancy at birth (years)	-0.17	< 0.001***
Extreme poverty	0.21	< 0.001***
Human development index	-0.01	< 0.001***
GDP per capita (current USD)	-0.09	< 0.001***
Diabetes prevalence (%)	0.13	< 0.001***
Cardiovascular death rate (%)	-0.02	0.401
Population aged 60 and above (%)	0.15	< 0.001***
Hospital beds per thousand people	-0.17	< 0.001***
Access to improved drinking water (%)	-0.30	< 0.001***
Prevalence of open defecation (%)	0.23	< 0.001***
Tourist/visitor arrivals (thousands per year)	-0.60	< 0.001***
Policy stringency index	0.25	< 0.001***

***P < 0.001

### Association of daily new cases with socio-economic and demographic factors and policy response

In the multivariable GAM model, a 1 unit (people per km^2^) increase in population density was associated with a 5.17% (lower confidence interval 5.11%, upper confidence interval 5.23%) growth in daily new COVID-19 cases. In addition, daily new COVID-19 cases (all *P* < 0.001) were correlated with life expectancy at birth (-1.61%), extreme poverty (8.95%), human development index (-0.05%), GDP per capita (-0.18%), diabetes prevalence (0.66%), proportion of population aged 60 and above (2.23%), hospital beds per thousand people (-0.08%), proportion of people with access to improved drinking water (-7.40%), prevalence of open defecation (0.69%), annual tourist/visitor arrivals (0.003%) and policy stringency index (1.95%), after controlling other potential confounders (all *P* < 0.001; Table 3).

**Table 3:** Estimated effects of socio-economic and demographic characteristics, health vulnerability and resources, and policy response on daily new COVID-19 cases in BRICS countries

Variables	Daily new cases			
	β	Lower confidence interval	Upper confidence interval	*P*-value
Policy stringency index	1.95%	1.70%	2.19%	< 0.001***
Population density (people per km^2^)	5.17%	5.11%	5.23%	< 0.001***
Life expectancy at birth (years)	-1.61%	-1.87%	-1.36%	< 0.001***
Extreme poverty	8.95%	7.85%	10.04%	< 0.001***
Human development index	-0.05%	-0.06%	-0.04%	< 0.001***
GDP per capita (current USD)	-0.18%	-0.20%	-0.16%	< 0.001***
Diabetes prevalence (%)	0.66%	0.58%	0.74%	< 0.001***
Population aged 60 and above (%)	2.23%	1.96%	2.50%	< 0.001***
Hospital beds per thousand people	-0.08%	-0.09%	-0.06%	< 0.001***
Access to improved drinking water (%)	-7.40%	-8.35%	-6.46%	< 0.001***
Prevalence of open defecation (%)	0.69%	0.61%	0.77%	< 0.001***
Tourist/visitor arrivals (thousands per year)	0.00%	0.00%	0.00%	< 0.001***

***P < 0.001

Different lag structures also indicated similar results on the association of daily new COVID-19 cases with socio-economic and demographic disadvantages, health and resource vulnerabilities, population mobility, and policy response in the sensitivity analysis (all *P* < 0.001).

**Fig. 2:**
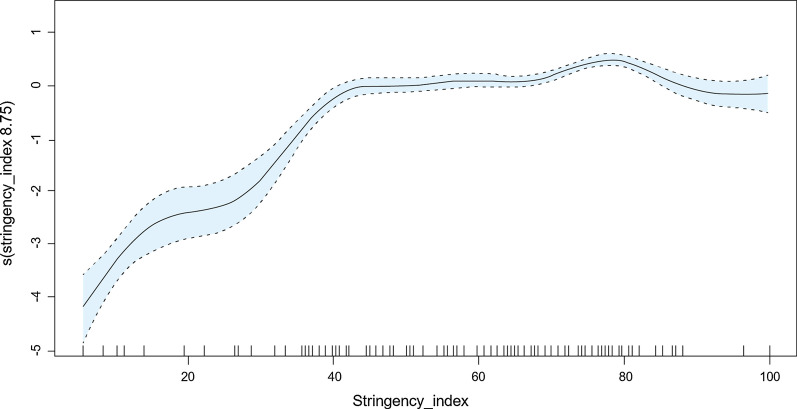
Exposure–response curves on the effects of policy stringency index in daily new COVID-19 cases. The x-axis represents the policy stringency index. The y-axis shows the contribution of the smoother to the fitted values

The exposure–response curves showed that the number of daily new COVID-19 cases increased with the rise in policy stringency index. There was a rapid surge in number of daily new COVID-19 cases when the policy stringency index ranged from 0 to 45. The number of infections climbed slowly when the policy stringency index ranged from 46 to 80, and decreased when the index was above 80, after controlling other potential confounders (Fig. 2).

## Discussion

To the best of our knowledge, this is the first study that evaluated the association of daily new COVID-19 cases with socio-economic and demographic factors, health vulnerability, resources, and policy response to the pandemic in BRICS countries. Our findings indicated that socio-economic and demographic factors, health and resource vulnerabilities, population mobility, and policy responses were associated with the COVID-19 pandemic. All results of the GAM model remained stable in the sensitivity analysis. After controlling the effect of other potential confounders, daily new cases had a positive relationship with the policy stringency index when the latter was below 80. In contrast, there was a negative relationship between daily new COVID-19 cases and the policy stringency index when the index reached above 80. These results indicated that strong policy response at the national level is crucial to reduce COVID-19 infections and control the pandemic.

Previous findings on the associations among socio-economic and demographic factors, health vulnerability, resources, and COVID-19 policy response were contentious. Rocha et al. [6] found that the initial spread of COVID-19 infections in the country was most affected by patterns of socio-economic vulnerability rather than population age structure and prevalence of existing chronic diseases. Kayral et al. [20] reported the association between the policy stringency and total cases in E7 countries during the COVID-19 period. In this study, we also found that countries with the most disadvantaged communities, vulnerable health system, and less resources (including health system as well water and sanitation) were associated with more COVID-19 cases in BRICS countries. Our results were consistent with previous findings [6, 20]. It supported the evidence that socio-economic vulnerability might have impact on the disproportionate burden on states and municipalities.

We found that a 1 unit increase in population density and extreme poverty were associated with a 5.17% and 8.95% growth in daily new COVID-19 cases respectively. Furthermore, a 1 percent rise in the proportion of population aged 60 and above and diabetes prevalence were associated with a 2.23% and 0.66% increase in daily new COVID-19 cases, respectively. One possible explanation was that the macro socio-economic environment and health system resilience at the national level had a higher impact on the pandemic than individual level, because the impact of individual health vulnerability might be affected by the possibility of exposure to the virus [25]. Socio-economic and demographic factors, health system vulnerability, as well as lack of water and sanitation resources could reflect the macro environment and system resilience. Mujica et al. found that socio-economic inequalities were associated with health outcomes (mortality) in BRICS countries [21]. Our findings highlighted the importance of reducing socio-economic inequalities and strengthening the resilience of health systems to better respond to public health emergencies in the future.

Brazil, Russia, India, China, and South Africa had nearly 45% of the world’s population [11]. Comparative studies on BRICS countries’ health outcomes (such as mortality of chronic diseases) had been conducted but there were no studies focusing on COVID-19. Romaniuk et al. [11] compared the health system outcomes of BRICS countries in 2000−2017 and found that Russia, China, and Brazil had better healthcare performance as opposed to India and South Africa. However, the entire group’s overall healthcare performance did not fare well compared to developed countries. They also found that the health system outcomes (such as infant mortality rate and maternal mortality rate) appeared to correlate on a statistically significant scale with GDP per capita [11]. During the COVID-19 pandemic, India, Brazil, and Russia were three of the top five countries with the highest disease burden of COVID-19, along with the United States and France. Moreover, the cumulative total COVID-19 cases in BRICS countries accounted for more than a quarter (26.3%) of the global total as of April 30, 2021.

Each BRICS country has its specific characteristics in terms of health performance and policy response to COVID-19 [3–5, 9, 12, 26, 27]. For instance, China had the largest total population with high population mobility and faced significant challenges on COVID-19 prevention and control. China adopted stringent policies and containment strategies, including active case detection and management, lockdown, intercity travel ban, practicing physical distancing and personal protection measures (including hand hygiene, face mask use, etc.). China managed to control its COVID-19 outbreak within three months. While China had the highest absolute number of vaccinations among BRICS countries, it still faced challenges on the relatively low level of vaccination coverage due to its large population. On the other hand, India faced the biggest challenge in coping with COVID-19 among BRICS countries, given its healthcare system vulnerability and high disease burden of COVID-19 as well as low immunization coverage.

There are some limitations in our study. First, COVID-19 and related indicators data were based on the national level instead of provincial or city level. We were not able to conduct analysis at provincial or city level. Second, some indicators (such as daily coverage of PCR test on SARS-CoV-2, daily vaccination coverage) were not included in the GAM model due to unavailable data. Third, bilateral correlations were only done in BRICS countries and statistical significance might be underpowered because of limited countries included.

## Conclusions

In BRICS countries, daily new COVID-19 cases were affected by socio-economic and demographic status, health vulnerabilities, resources, and policy response. It is important to reduce socio-economic inequalities and strengthen the resilience of health systems to better respond to public health emergencies. Future studies are needed to explore the way of strengthening public health and health systems resilience in all countries to utilize interventions on curbing the pandemic effectively.

## Data Availability

The datasets generated during and/or analyzed during the current study are available in the Our world in Data (https://ourworldindata.org/coronavirus-source-data), WHO (https://covid19.who.int/), and UN (http://data.un.org/).

## Abbreviations

COVID-19: Coronavirus disease 2019
SARS-CoV-2: Severe acute respiratory syndrome coronavirus
BRICS: Brazil, Russia, India, China, and South Africa
WHO: World Health Organization
UN: United Nations
GAM: Generalized Additive Model
